# Excess daytime sleepiness, daily functioning and health-related quality of life during the first year after metabolic and bariatric surgery in patients with a known and unknown obstructive sleep apnea status

**DOI:** 10.1007/s11325-026-03729-5

**Published:** 2026-06-15

**Authors:** Claudia Berends, Laura Heusschen, Sophie L. van Veldhuisen, Steve M. M. de Castro, Ruben N. van Veen, Marinus J. Wiezer, Ronald S. L. Liem, François M. H. van Dielen, Ahmet Demirkiran, Evert-Jan G. Boerma, Timon M. Fabius, Eric J. Hazebroek

**Affiliations:** 1 Vitalys Obesity Clinic part of Rijnstate Hospital, Arnhem, The Netherlands; 2 Division of Human Nutrition and Health, Wageningen University & Research, Wageningen, The Netherlands; 3 Department of Surgery, Onze Lieve Vrouwe Gasthuis, Amsterdam, The Netherlands; 4 Department of Surgery, St. Antonius Hospital, Nieuwegein, The Netherlands; 5 Dutch Obesity Clinic, The Hague, The Netherlands; 6 Department of Surgery, Máxima Medical Center, Veldhoven, The Netherlands; 7 Department of Surgery, Rode Kruis Hospital, Beverwijk, The Netherlands; 8 Department of Surgery, Zuyderland Medical Center, Heerlen, The Netherlands; 9 Department of Pulmonology, Medisch Spectrum Twente, Enschede, The Netherlands

**Keywords:** Obstructive sleep apnea, Excess daytime sleepiness, Health related quality of life, Metabolic, Bariatric surgery

## Abstract

**Purpose:**

Despite a high prevalence of obstructive sleep apnea (OSA) in patients undergoing metabolic and bariatric surgery (MBS), there is no consensus on the best perioperative OSA strategy. Supplemental oxygen therapy without pre-operative OSA-testing (pulse oximetry) is safe and more cost-effective compared to preoperative OSA-testing using poly(somno)graphy (sleep test). This study aimed to compare these strategies on excess daytime sleepiness (EDS), its impact on daily functioning and health-related quality of life (HRQoL).

**Methods:**

Multicenter prospective cohort study including 1388 patients, 691 in the sleep test and 697 in the pulse oximetry group based on local protocols. EDS (ESS), its impact on daily functioning (FOSQ-10), HRQoL (RAND-36) and total weight loss (TWL) were assessed preoperatively and 1, 3, 6 and 12 months postoperatively. (Sub)scores were compared over time between and within groups using linear mixed models.

**Results:**

No differences between the pulse oximetry and sleep test strategy were found in EDS, its influence on daily life and HRQoL, and all improved after MBS. In the pulse oximetry group a lower TWL was found 1 month postoperative (-0.7%).

**Conclusion:**

Considering that pulse oximetry is more cost-effective, equally safe and comparable in EDS, its effect on daily functioning and HRQoL and no clinically relevant difference in TWL was found compared to the sleep test strategy, this study lends further support for pulse oximetry being the best strategy for managing OSA in patients undergoing MBS.

**Supplementary Information:**

The online version contains supplementary material available at 10.1007/s11325-026-03729-5.

## Introduction

Metabolic and bariatric surgery (MBS), is an effective treatment against obesity. In patients undergoing MBS, the prevalence of obstructive sleep apnea (OSA) is high, (35–95%), and mostly undiagnosed [1]. Patients with OSA have a potentially increased risk of postoperative cardiac events, respiratory failure and admission to the intensive care unit (ICU) [2, 3]. Despite the high prevalence and increased risk of postoperative complications, there is still no consensus for the best perioperative strategy to manage OSA in patients undergoing MBS.

In the Netherlands, there are currently two strategies that are mainly used. In the first strategy, patients are tested for OSA preoperatively by overnight poly(somno)graphy, and if indicated treated with positive airway pressure (PAP), referred to as the sleep test strategy. In the second strategy, patients are not tested for OSA, but receive supplemental oxygen therapy and are postoperatively continuously monitored with pulse oximetry (pulse oximetry strategy). The pulse oximetry strategy is based upon the hypothesis that losing weight itself can improve or resolve OSA in the long term [4, 5]. Perioperative safety is guaranteed by continuous monitoring, without the additional costs and use of healthcare resources for testing and (temporarily) treating bariatric patients for OSA preoperatively.

The cost-effectiveness and patient safety of these two strategies was compared in a large prospective cohort study (POPCORN study), and found no differences in post-operative complications and unscheduled intensive care unit admissions between the two strategies, and the health-related costs were lower for the pulse oximetry strategy (-€534 per patient) [6].

In addition to patient safety and cost-effectiveness, the impact of both strategies should also focus on perhaps the most important outcomes for patients potentially suffering from OSA; excess daytime sleepiness (EDS), and related daily functioning and health-related quality of life (HRQoL). In the pulse oximetry group, part of the patients will have untreated OSA, given that it will take several months to lose sufficient weight for OSA to improve, these patients may experience more EDS during this first postoperative period. However, both obesity and OSA are found to be risk factors for EDS, and previous research showed a significant postoperative decrease in EDS in both bariatric patients with and without OSA [7–9]. This had led to the hypothesis that having untreated OSA will not influence postoperative EDS following MBS.

Therefore, the aim of this study was to compare two perioperative strategies for OSA (sleep test and pulse oximetry strategy) on post-operative EDS and its impact on daily functioning and HRQoL before and after MBS.

## Methods

### Study design

This research was conducted as part of the POPCORN study, a multicenter prospective cohort study among patients undergoing MBS. Recruitment took place at seven hospitals in the Netherlands (Rijnstate hospital, Arnhem; St. Antonius Hospital, Nieuwegein; Onze Lieve Vrouwe Hospital, Amsterdam; Dutch Obesity Clinic, the Hague; Zuyderland Hospital, Heerlen; Rode Kruis Hospital, Beverwijk and Máxima Medical Centre, Veldhoven) between April 2018 and February 2020. Patients undergoing primary laparoscopic Roux-en-Y gastric bypass (RYGB) or laparoscopic sleeve gastrectomy (SG) at one of the participating centers were eligible for this study. Exclusion criteria were prior MBS, prior OSA diagnosis or poly(smono)graphy, undergoing concomitant surgical procedures (e.g. cholecystectomy) and the inability to speak or read the Dutch language. Detailed information on the study design can be found in the previously published study protocol [10].

All participants gave written informed consent for their participation in this study. The study was approved by the Medical research Ethics Committees United (MEC-U reference number W17.050), and adheres to the Declaration of Helsinki.

Participants received usual care as per local protocol, which included a one-night postoperative hospital stay. Beyond usual care, patients were asked to complete several questionnaires, among which the Epworth sleepiness scale (ESS), functional outcome of sleep questionnaire (FOSQ-10), and RAND-36 pre-operatively and one, three, six and twelve months after surgery.

Participants in the pulse oximetry group were only enrolled at Rijnstate Hospital, as this was the only hospital among the participating centers that used this strategy. In this group, patients were not preoperatively tested for OSA. Postoperatively, patients were continuously monitored using pulse oximetry and received supplemental oxygen therapy through a nasal cannula (2 L/min). Nurses were alerted in case of desaturations (peripheral oxygen saturation [SpO_2_] < 90%). In case of persisting desaturations, nurses intervened by waking up the patient and/or increasing oxygen flow. If these interventions were insufficient, the attending physician was informed for clinical evaluation and treatment. In the sleep test group, all patients were preoperatively tested for OSA by ambulatory P(S)G and if indicated PAP treatment was started.

### Data collection

The following information was obtained from electronic patient records: age, gender, body mass index (BMI), waist circumference, OSA diagnosis (diagnosed with poly(somno)graphy), occurrence of obesity related comorbidities (hypertension, dyslipidemia, diabetes mellitus type 2), occurrence of OSA related comorbidities and events (asthma, chronic obstructive pulmonary disease (COPD), myocardial infraction, cerebral vascular accident (CVA), type of surgery (RYGB, SG), total weight loss (preoperative weight - current weight)/(preoperative weight) x 100), smoking status (categorized as: smoker, former smoker, non-smoker). Primary outcomes were EDS, the impact of EDS on daily functioning and HRQoL.

### Excess daytime sleepiness

EDS was determined using the Epworth sleepiness scale (ESS). The ESS consists of eight questions in which the patient has to rate, on a scale of 0 to 3, how high the chance is of dozing off or falling asleep in different situations. The ESS score equals the sum of these eight ratings, giving a score between 0 and 24. If one or more of the eight ratings are missing, the ESS score cannot be calculated. Low scores indicate a low degree of sleepiness, an ESS score of > 10 or higher indicates excessive daytime sleepiness [11, 12].

### Impact of EDS on daily functioning

The impact of EDS on functioning in daily life was assessed by FOSQ-10 [13]. The version of the FOSQ-10 used in this study can be found in Supplementary Material 1. The score of the FOSQ-10 ranges from 5 to 20 points, a higher score indicating better functioning in daily life. If there were one or more missing answers, the score for FOSQ-10 was not calculated.

### Health-related quality of life

HRQoL was assessed with the RAND-36 questionnaire. This questionnaire consists of 36 questions within nine subscales: physical functioning, role limitations due to physical health, role limitations due to emotional problems, energy/fatigue, emotional well-being, social functioning, pain, general health, and health change. The score for each subscale ranges from 0 to 100, with higher scores defining a better quality of life [14, 15]. If one or more answers were missing for a subscale, the score for that subscale was not calculated.

### Statistical analysis

Based on normality, data were expressed as mean with standard deviation (normal data), or median with Q1-Q3 (non-normal data). Patient characteristics were compared between the groups using independent T-tests (normally distributed, continuous data), Mann-Whitney U tests (not normally distributed continuous data) or Chi Square tests (binary data).

The (sub)scores of the ESS, FOSQ-10, RAND-36 and total weight loss were compared over time between the pulse oximetry and sleep test group using linear mixed models. Prior to analysis, the FOSQ-10 and the RAND-36 sub scores physical functioning, emotional well-being, social functioning, pain and health change were reflected and then square-rooted, for the ESS score square root transformation without reflection was applied. The model consisted of fixed effects for timepoint (preoperative and 1, 3, 6 and 12 months postoperative) and group (pulse oximetry; sleep test), the interaction between timepoint and group and a random effect for participants. Age, BMI and gender were added as covariates, as these were found to be confounders. If a significant difference was found, post-hoc pairwise comparisons were done within the pulse oximetry and sleep test group for all questionnaires at each timepoint. The Bonferroni correction was applied to correct for multiple comparisons in the post-hoc pairwise comparisons. Data is presented as estimated marginal means with upper and lower range. All data were analyzed using SPSS 29.0 for Windows. A p-value below 0.05 was considered statistically significant.

## Results

A total of 1432 patients were enrolled of whom 44 patients were excluded due to not undergoing the metabolic bariatric procedure (*n* = 28), undergoing concomitant surgical procedures (*n* = 12), missing pre-operative sleep test data (*n* = 2), or PAP treatment within the pulse oximetry strategy (*n*=2). This resulted in a total study population of 1388 patients: 697 in the pulse oximetry group and 691 in the sleep test group. Participants in the pulse oximetry group were less often men (18.2% versus 23.3%, *p*=.02), were younger (43.7 ± 0 versus 45.6 ± 11.6 years, *p*=.03), more often underwent RYGB (79.5% vs. 74.7%, *p*=.03), exhibited a lower prevalence of hypertension (36.2% versus 42.5%, *p*=.02), and dyslipidemia (20.5% versus (30.0%, *p*<.001) before surgery compared to the pulse oximetry group (Table 1).

**Table 1:** Patient characteristics for the total study population and in the pulse oximetry (unknown OSA status) and sleep test (known OSA status) group before MBS

Characteristic	All (*n* = 1388)	Pulse oximetry (*n* = 697)	Sleep test (*n* = 691)	*p*
Gender (male)	288 (20.7)	127 (18.2)	161 (23.3)	0.02*
Age (years)	44.6 ± 11.9	43.7 ± 12.0	45.6 ± 11.6	0.003*
Body Mass Index (kg/m^2^)	42.3 [39.8–46.3]	42.5 [39.9–46.7]	41.9 [39.8–45.8]	0.08
Waist circumference (cm)	127 [119–137]^1^	127 [119–136]^2^	127 [119–137]^3^	0.84
OSA diagnosis	n/a	n/a	338 (48.9)	
Obesity related comorbidities				
Hypertension	546 (39.3)	252 (36.2)	294 (42.5)	0.02*
Dyslipidemia	350 (25.2)	143 (20.5)	207 (30.0)	< 0.001*
Diabetes mellitus type 2	245 (17.7)	113 (16.2)	132 (19.1)	0.17
OSA related comorbidities and events				
Asthma	172 (12.5)^4^	91 (13.4)^5^	81 (11.7)	0.36
COPD	37 (2.7)^6^	20 (2.9)^7^	17 (2.5)	0.58
Myocardial infarction	38 (2.7)^8^	17 (2.4)^9^	21 (3.0)^10^	0.50
Cerebral vascular accident	14(1.0)^11^	8 (1.1)	6 (0.9)^12^	0.60
Smoking				
Non-smoker	841 (60.6)	424 (60.8)	417 (60.3)	0.30
Smoker	104 (7.5)	59 (8.5)	45 (6.5)	
Former smoker	443 (31.9)	214 (30.7)	229 (33.1)	
Type of surgery				
RYGB	1070 (77.1)	554 (79.5)	516 (74.7)	0.03*
SG^13^	318 (22.9)	143 (20.5)	175 (25.3)	

Data are presented as mean ± SD, median [Q1-Q3] or frequency (percentage), ^1^ *n* = 1291, ^2^ *n* = 623, ^3^ *n* = 668,^4^ *n* = 1372, ^5^ *n* = 681, ^6^ *n* = 1369,^7^ *n* = 678, ^8^=1385, ^9^*n* = 696, ^10^*n* = 689, ^11^*n* = 1387, ^12^*n* = 690, ^13^ one partial sleeve gastrectomy, pulse oximetry: participants with postoperative continuous pulse oximetry, sleep test: participants with preoperative polysomnography, *OSA* obstructive sleep apnea, *COPD* chronic obstructive pulmonary disease, *RYGB* Roux-and Y Gastric Bypass, *SG* laparoscopic sleeve gastrectomy

### Excess daytime sleepiness

**Fig. 1:**
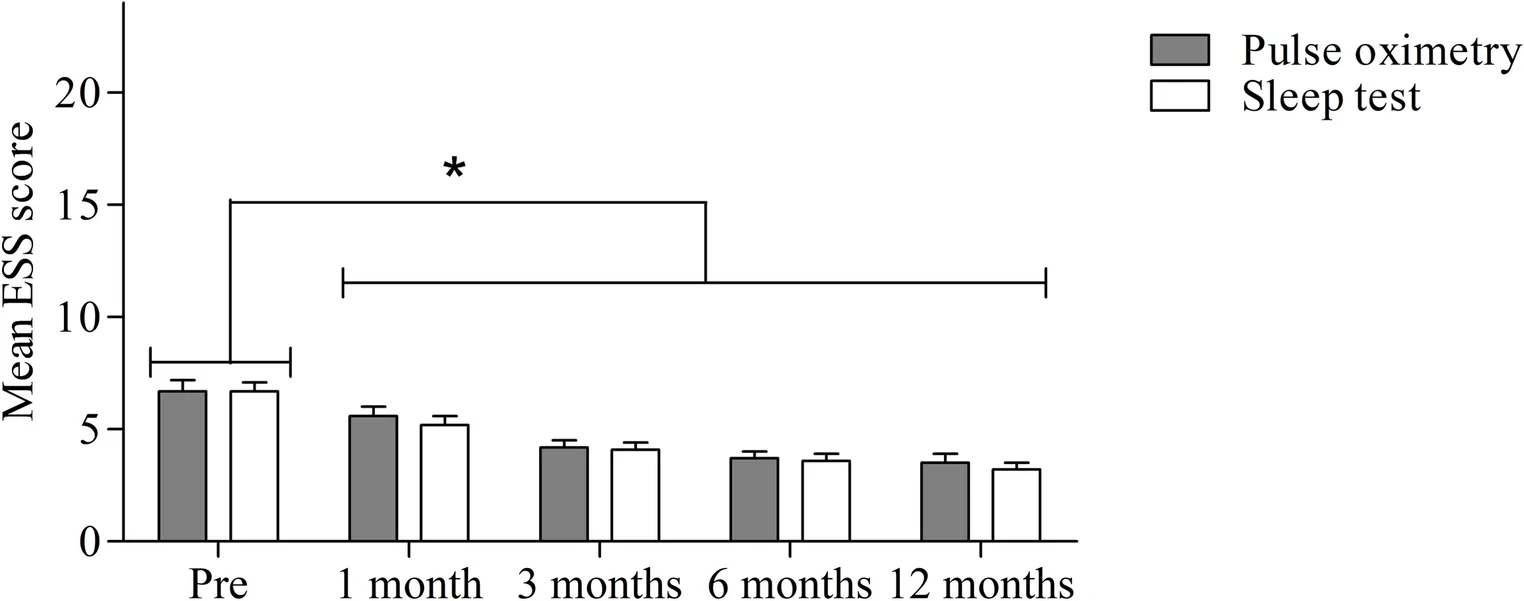
Estimated marginal mean ESS scores before and after MBS for the pulse oximetry group and sleep test group *significant improvement of the score within the group (pulse oximetry or sleep test) compared to pre-operative (*p*<.001)

ESS scores before surgery and during follow-up were similar in the pulse oximetry and sleep test group (*p*=.24). In both groups, a significant improvement in ESS score was seen from 1 month post-operatively onwards (*p*<.001). In the pulse oximetry group, the mean score improved from 6.7 [95% CI: 6.2 to 7.2] pre-operatively to 3.5 [95% CI: 3.2 to 3.9] at 12 months post-operative and in the sleep test group from 6.7 [95% CI: 6.2 to 7.1] to 3.2 [95% CI: 2.8 to 3.5] (Fig. 1). Pre-operatively, 25.2% of the patients in the pulse oximetry group and 26.4% in the sleep test group had an ESS > 10 (*p*=.62). This improved in both groups to respectively 10.2% and 10.6% at 12 months after surgery (*p*=.83). Detailed data of the ESS scores and number of participants at each time point can be found in Supplementary Table 1.

### Impact of EDS on daily functioning

**Fig. 2:**
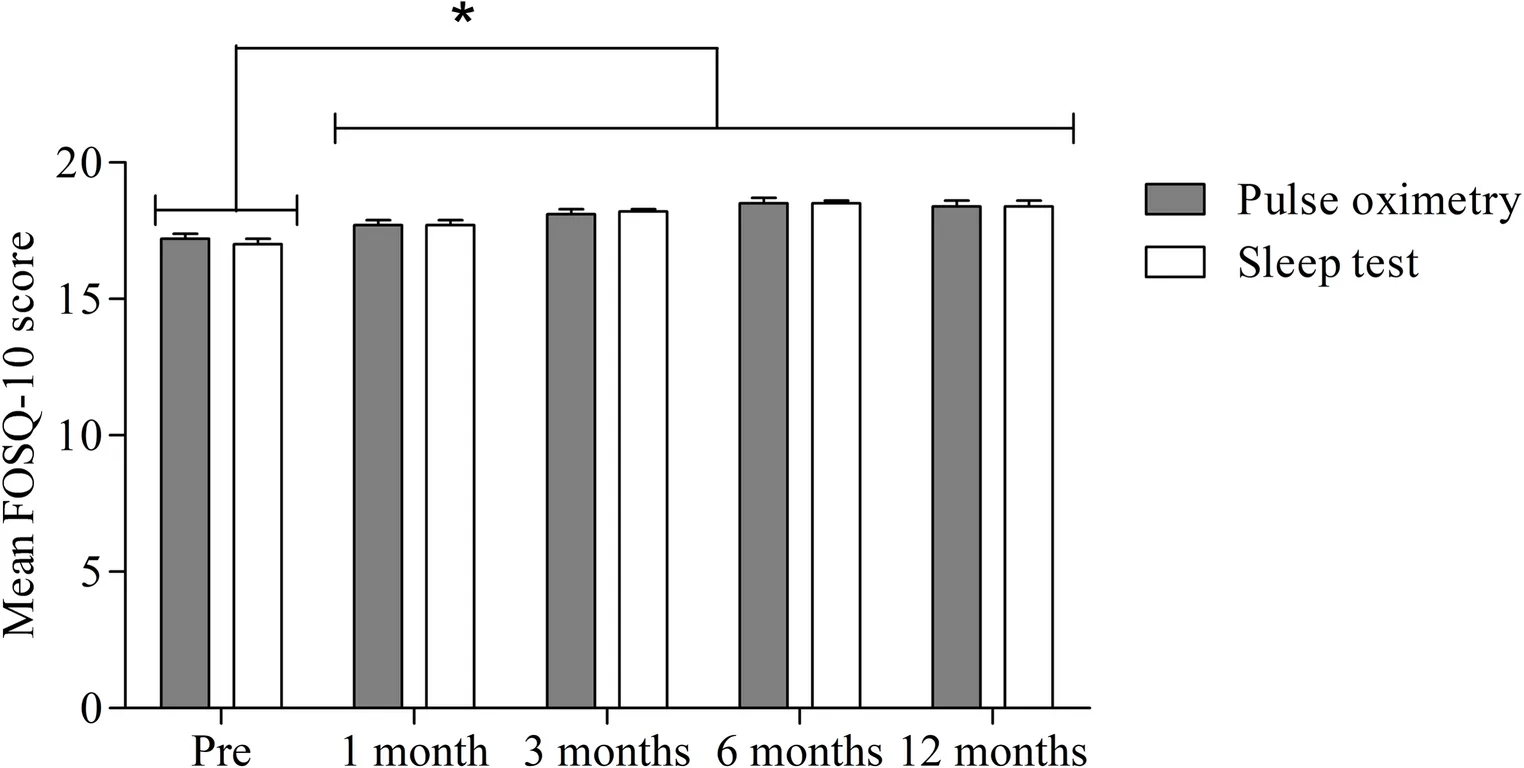
Estimated marginal mean FOSQ-10 scores before and after MBS for the pulse oximetry group and sleep test group *significant improvement of the score within the group (pulse oximetry or sleep test) compared to pre-operative (*p*<.001)

FOSQ-10 scores were comparable before surgery and during follow up in the pulse oximetry and sleep test group (*p*=.68). Within both groups, a significant improvement in the FOSQ-10 score was seen from 1 month post-operatively onwards (*p* <.001). The mean score improved from 17.2 [95% CI: 16.9 to 17.4] pre-operatively to 18.4 [95% CI: 18.2 to 18.6] at 12 months post-operatively in the pulse oximetry group and from 17.0 [95% CI: 16.8 to 17.2] to 18.4 [95% CI: 18.2 to18.6] in the sleep test group (Fig. 2). Detailed data of the FOSQ-10 scores and number of participants at each time point can be found in Supplementary Table 2.

### HRQoL

**Fig. 3:**
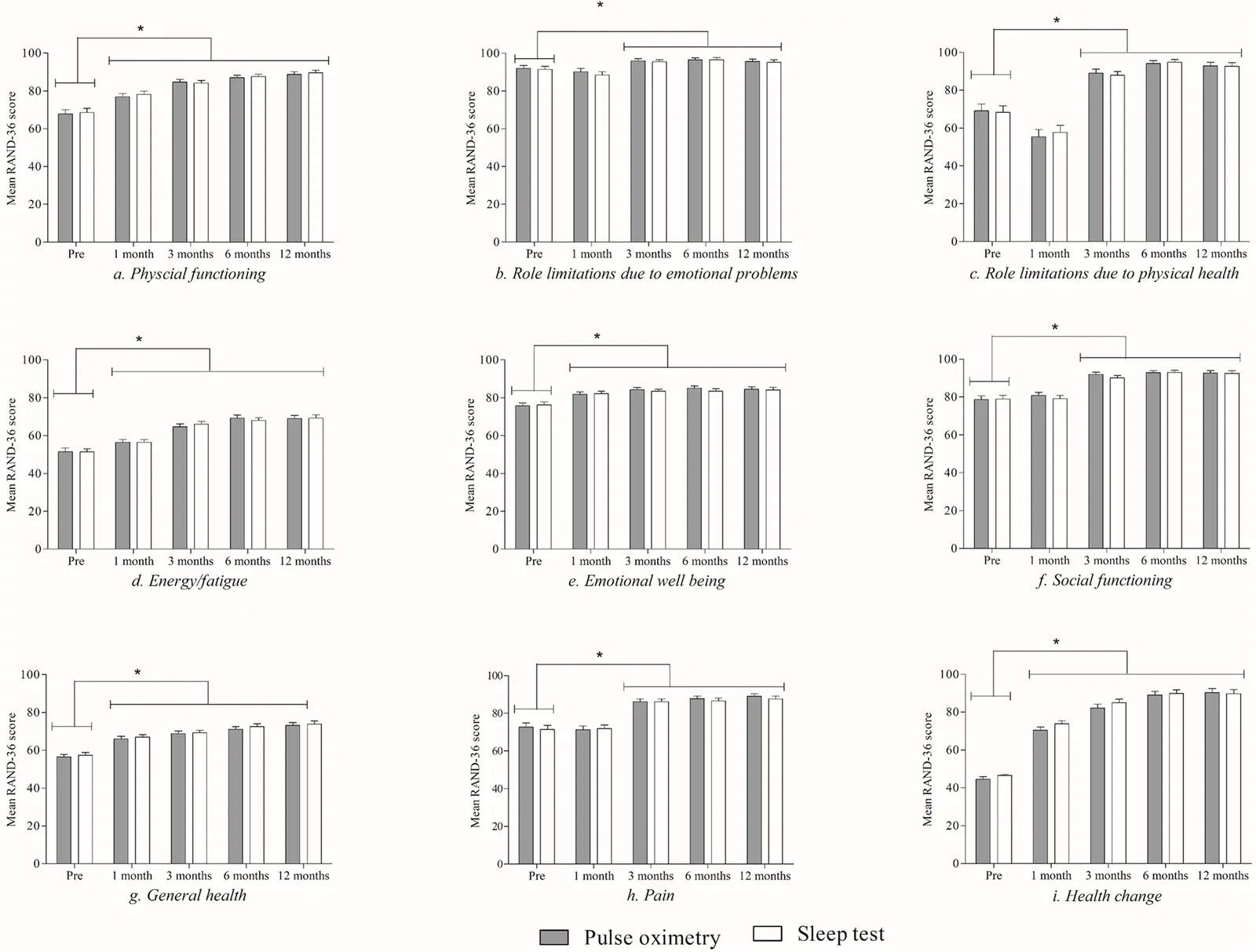
Estimated marginal mean ean RAND-36 score for each category before and after MBS for the pulse oximetry group and sleep test group *significant improvement of the score within the group (pulse oximetry or sleep test) compared to pre-operative (*p*<.001)

The postoperative course of all nine categories of the RAND-36 was similar between the pulse oximetry and sleep test group (*p*>.05). Within both groups, RAND-36 scores of all subscales/categories improved after surgery. This significant improvement was seen from 1 month onwards in the categories physical functioning, energy/fatigue, emotional well-being, general health and health change (*p* <.001; Fig. 3a, d, e, g and i), and from 3 months onwards in the categories social functioning and pain (*p* <.001; Fig. 3f and h). In the categories role limitations due to emotional problems and role limitations due to physical health, there was a deterioration in scores at 1 month post-operatively, followed by an improvement from 3 months onwards (Fig. 3b and c). The deterioration pre-operatively to 1 month postoperatively was for role limitations due to emotional problems from 91.8 [95% CI: 90.0 to 93.5] to 90.1 [95%CI: 88.3 to 91.8] in the pulse oximetry group and 91.4 [95% CI: 89.6 to 93.0] to 88.4 [95% CI: 86.4 to 90.2] in the sleep test group, and for role limitations due to physical health from 69.2 [95% CI: 65.5 to 72.7] to 55.4 [95% CI: 51.4 to 59.2] in the pulse oximetry group and 68.3 [95% CI: 64.6 to 71.8] to 57.8 [95% CI: 53.9 to 61.5] in the PG group. Detailed data of the RAND-36 scores and number of participants at each time point can be found in Supplementary Table 3.

### Total weight loss

**Fig. 4:**
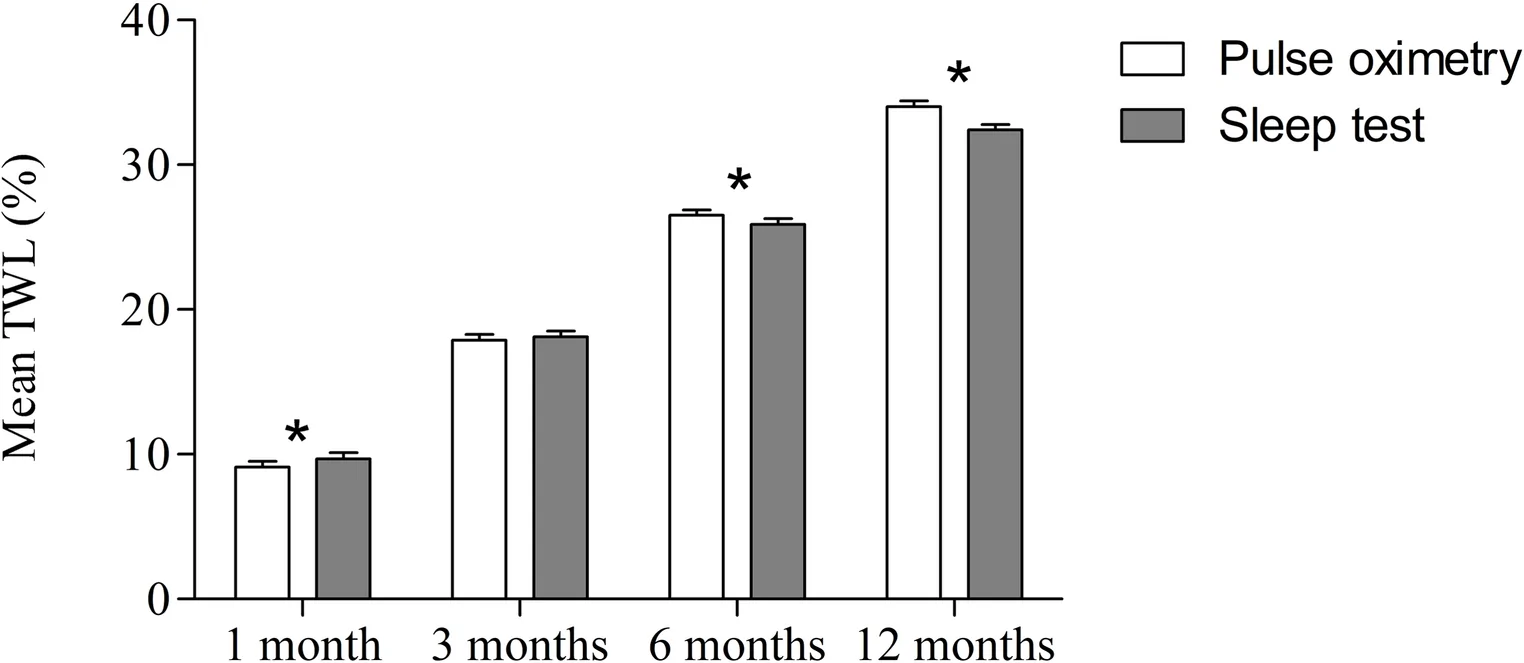
Estimated marginal mean total weight loss (%) after MBS for the sleep test strategy and pulse oximetry strategy *significant difference between the pulse oximetry and sleep test group (*p*<.05), *TWL* total weight loss

The postoperative course of total weight loss was different between the pulse oximetry and sleep test strategy (Fig. 4). Post-hoc comparisons showed a significantly lower total weight loss in the pulse oximetry group 1 month after surgery (9.0% [95%CI: 8.7 to 9.5] versus 9.7 [95%CI: 9.3 to 10.1]; *p*=.03), and a higher total weight loss at 6 months (26.5 [95%CI: 26.0 to 26.9] versus 25.9 [95%CI: 25.5 to 26.3]; *p*=.04) and 12 months after MBS (34.0 [95%CI: 33.6 to 34.4] versus 32.4 [95% CI: 32.0 to 32.8], p = < 0.001), when compared to the sleep test group. Within the strategies, total weight loss increased significantly from 1 month postoperative onwards (*p*<.001). Estimated marginal means of total weight loss percentage and number of participants at each time point can be found in Supplementary Table 4.

## Discussion

The aim of this study was to compare two perioperative strategies for OSA (sleep test and pulse oximetry) on post-operative EDS and its impact on functioning in daily life, HRQoL and total weight loss after MBS. We found no significant difference between the pulse oximetry and sleep test group in EDS, impact of EDS on daily life and HRQoL and all of these outcomes significantly improved during the first 12 months after MBS. In the pulse oximetry group, a lower total weight loss was found 1 month postoperative (−0.7%) and a higher total weight loss at 6 and 12 months postoperative (+ 0.6% and + 1.6%), compared to the sleep test strategy.

In our study, EDS improvement was shown with increased ESS scores of 3.2 (pulse oximetry) and 3.5 (sleep test) points at 12 months after surgery. This meets the Minimum Clinically Important Difference (MCID) for ESS in OSA patients, which is set to an improvement of at least 2–3 points [16, 17]. Similarly, the prevalence of patients with an ESS score > 10 decreased with 15.0% in the pulse oximetry group and 15.8% in the sleep test group after 12 months. This is comparable to two previous studies, the first study being performed in a population undergoing RYGB with a preoperative OSA prevalence of 71% and the second study in a population with unknown OSA status undergoing bariatric surgery [4, 18]. In these studies, ESS scores improved with 3.1 and 2.3 points and a decrease of 22% and 20% was seen in ESS ≥ 10 at 12 and 16 months after surgery.

Two other studies found a higher improvement in ESS score, i.e. 6.0 points 5 months after SG in a population with unknown pre-operative OSA status and 4.9 points 12 months after SG or RYGB in a population with a pre-operative OSA prevalence of 96.3%. However, in these studies sample sizes were small, respectively 13 and 27 patients [19, 20].

The reason we did not find a difference in EDS between the pulse oximetry and sleep test group, may be because EDS is not always seen in patients with OSA, and EDS is not only associated with OSA [7, 21]. In two aforementioned studies in an MBS population, a pre-operative prevalence of OSA of 71% and 96.3% was found, while EDS was only found in 32.0% and 29.6% of the patients [4, 20]. In two other studies comparing participants with obesity to normal weight participants, both without OSA, a significant higher prevalence of EDS was found in patients with obesity, respectively 57% versus 1% and 34.7% versus 2.7% [22, 23]. In a cross-sectional study, BMI was independently associated with EDS (*p* <.001); in fact, the authors found that depression and metabolic factors (such as obesity) were more strongly associated with EDS than sleep disordered breathing or sleep disturbances [21].

The impact of EDS on daily functioning improved from 1 month postoperatively onwards with an improvement of 1.2 points in FOSQ-10 score in the pulse oximetry group and 1.4 point in the and sleep test group, 6 months after surgery. This is below the MCID, which is set to an increase of 1.8–2.2 points in patients with OSA [24]. This is probably due to the already high pre-operative scores, i.e. 17.2 [95% CI:16.9 to 17.4] in the pulse oximetry and 17.0 [95% CI: 16.8 to 17.2] in the sleep test group on a scale of 5–20, leaving little room for improvement. To our knowledge, one other study compared the influence of EDS on daily life after MBS. This study found a slightly better improvement of 1.9 (non-OSA) and 2.3 (OSA) points, 6 months after surgery [9]. However, sample sizes were small with respectively 40 (non-OSA) and 50 (OSA) patients.

We found a significant improvement in HRQoL after MBS, which is consistent with previous research measuring HRQoL after MBS [25–28]. We did not find a difference in HRQoL between the pulse oximetry and sleep test group, probably because weight loss itself after MBS improves HRQoL [29]. Total weight loss was different during the first year after MBS between the pulse oximetry and sleep test strategy. One month after surgery, total weight loss was lower in the pulse oximetry group, after 3 months weight loss was comparable and from 6 months onwards the weight loss was higher in the pulse oximetry strategy compared to the sleep test strategy. This might be explained by the fact that reduced sleep quality and EDS, both associated with OSA, are known to impair weight loss after MBS [30, 31]. Additionally, OSA is known to improve 3–6 months after MBS, as participants in the pulse oximetry group may have untreated OSA, this could potentially explain the reduced weight loss observed during the early postoperative period [32]. Nonetheless, this does not explain the higher weight loss from 6 months postoperative onwards in the pulse oximetry group. This might be due to differences in the postoperative life style trajectory, as this is essential for weight loss results and all participants in the pulse oximetry group were included from one bariatric center, while the participants in the sleep test strategy were included in six centers. However, it must be noted that the maximum difference in weight loss was 1.6% (12 months after MBS). This difference is not considered clinically relevant as the absolute difference will only be 1–2 kg, and is not expected to influence HRQoL or EDS, as confirmed by the results in our study.

Strengths of this study were the large population and frequent follow up moments. However, there are some limitations that should be acknowledged. First, despite our best efforts to obtain complete questionnaires, there was a relatively high number of missing scores due to unanswered questions, probably because the questionnaires were self-reported by participants (number of participants for each time point can be found in Supplementary Tables 1, 2, 3 and 4). Second, there may be selection bias due to a center effect as some base line characteristics were different and all participants in the pulse oximetry group were included in a single bariatric center. We partially corrected this by using confounding factors (age, BMI, gender) in the linear mixed model analysis. Hypertension and dyslipidemia were not accounted for as these conditions are typically managed by medical treatment. Therefore, we do not anticipate a significant impact of hypertension and dyslipidemia on HRQoL outcomes. Third, the pre-operative prevalence of OSA in the pulse oximetry group and the postoperative prevalence of OSA in the total population are unknown. Therefore, it is not possible to conclude whether the changes in EDS, impact of EDS on daily life and HQoL are due to weight loss or less prevalent OSA.

## Conclusion

The present study found no differences in post-operative EDS and its impact on functioning in daily life and HRQoL 12 months after MBS, between patients with a known and unknown OSA status when comparing two perioperative strategies for OSA, i.e. pre-operative polysomnography and subsequently treatment with PAP if indicated (sleep test; known OSA status) to postoperative continuous monitoring by pulse oximetry and supplemental oxygen therapy without pre-operative testing for OSA (pulse oximetry; unknown OSA status). Considering that the pulse oximetry strategy is more cost-effective, equally safe and comparable in EDS, its effect on daily functioning and HRQoL and no clinically relevant difference in total weight loss was found compared to the sleep test strategy, this study lends further support for pulse oximetry being the best strategy for managing OSA in patients undergoing MBS. Future research is needed to show whether these results persist in long-term follow-up and to differentiate between the effects of weight loss and OSA prevalence.

## Supplementary Information

Below is the link to the electronic supplementary material.

Supplementary Material 1 (DOCX 41.8 KB)

## Data Availability

The data that support the findings of this study are available from the corresponding author upon reasonable request.
