# Supplementary material for: Excess daytime sleepiness, daily functioning and health-related quality of life during the first year after metabolic and bariatric surgery in patients with a known and unknown obstructive sleep apnea status
Source: Sleep Breath. 2026 Jun 15;30(4):188. doi: 10.1007/s11325-026-03729-5 (PMC13269512; doi:10.1007/s11325-026-03729-5)
Supplement: Supplementary file 1 — Supplementary Material 1 (DOCX 41.8 KB) [file 11325_2026_3729_MOESM1_ESM.docx]

Supplementary Material 1: Version of the FOSQ-10 used in the POPCORN study (Dutch version as used in the POPCORN study and the English translation).

**Dutch**

**FOSQ-10: Kwaliteit van leven met slaperigheid**

De komende vragen gaan over de relatie tussen de neiging om in slaap te vallen en de invloed daarvan op uw dagelijks leven.

Wij willen vragen om in de komende 10 situaties een score te geven wat het beste past bij uw leven.

Kies het getal dat het best past bij uw toestand in die situatie.

0= Ja, heel erg

1= Ja, behoorlijk

2= Ja, een beetje

3= Nee

0 1 2 3

| Heeft u moeite met het concentreren op alledaagse activiteiten omdat u moe of slaperig bent? |  |  |  |  |
| --- | --- | --- | --- | --- |
| Heeft u moeite met dingen onthouden omdat u moe of slaperig bent? |  |  |  |  |
| Heeft u moeite met het autorijden van korte afstanden (minder dan 160 km) omdat u dan slaperig wordt? |  |  |  |  |
| Heeft u moeite met het autorijden van korte afstanden (meer dan 160 km) omdat u dan slaperig wordt? |  |  |  |  |
| Heeft u moeite met op bezoek gaan bij familie of vrienden omdat u moe of slaperig wordt? |  |  |  |  |
| Is uw relatie met familieleden, vrienden of collega’s veranderd doordat u moe of slaperig bent? |  |  |  |  |
| Heeft u moeite met het kijken van een film omdat u slaperig of moe wordt? |  |  |  |  |
| Heeft u moeite om ‘s avonds net zo actief te zijn als u graag zou willen, omdat u slaperig of moe bent? |  |  |  |  |
| Heeft u moeite om ‘s ochtends net zo actief te zijn als u graag zou willen, omdat u slaperig of moe bent? |  |  |  |  |
| Wordt uw stemming beïnvloedt doordat u slaperig of vermoeid bent? |  |  |  |  |

**English
FOSQ-10: Quality of Life with Sleepiness**

The following questions are about the relationship between the tendency to fall asleep and its impact on your daily life.

We would like to ask you to give a score for the next 10 situations that best fits your life.

Please choose the number that best describes your condition in that situation.
0 = Yes, very much
1 = Yes, quite a bit
2 = Yes, a little
3 = No

0 1 2 3

| Do you have difficulty concentrating on the things you do because you are sleepy or tired? |  |  |  |  |
| --- | --- | --- | --- | --- |
| Do you have trouble remembering things because you are tired or sleepy? |  |  |  |  |
| Do you have trouble driving short distances (less than 160 km) because you become sleepy? |  |  |  |  |
| Do you have trouble driving long distances (more than 160 km) because you become sleepy? |  |  |  |  |
| Do you have trouble visiting family or friends because you become tired or sleepy? |  |  |  |  |
| Has your relationship with family members, friends, or colleagues changed because you are tired or sleepy? |  |  |  |  |
| Do you have trouble watching a movie because you become sleepy or tired? |  |  |  |  |
| Do you have trouble being as active in the evening as you would like because you are sleepy or tired? |  |  |  |  |
| Do you have trouble being as active in the morning as you would like because you are sleepy or tired? |  |  |  |  |
| Is your mood affected because you are sleepy or tired? |  |  |  |  |

.

Supplementary Table 1: Mean scores and number of patients that completed the ESS questionnaire at baseline and 1, 3, 6 and 12 months after surgery

|  | **Pulse oximetry % (n)** | **EMM [95% CI]** | **Sleep test % (n)** | **EMM [95% CI]** |
| --- | --- | --- | --- | --- |
| Baseline  1 month  3 months  6 months  12 months | 96.3 (671)  85.9 (599)  80.6 (562)  77.6 (541)  82.8 (577) | 6.7 [6.2 to 7.2]  5.6 [5.2 to 6.0]  4.2 [3.9 to 4.5]  3.7 [3.3 to 4.0]  3.5 [3.2 to 3.9] | 97.7 (675)  83.8 (579)  77.1 (533)  76.7 (530)  77.7 (537) | 6.7 [6.2 to 7.1]  5.2 [4.8 to 5.6]  4.1 [3.7 to 4.4]  3.6 [3.3 to 3.9]  3.2 [2.8 to 3.5] |

EMM: Estimated marginal mean, CI: Confidence Interval

Supplementary Table 2: Mean scores and number of patients that completed the FOSQ-10 questionnaire at baseline and 1, 3, 6 and 12 months after surgery

|  | **Pulse oximetry % (n)** | **EMM [95% CI]** | **Sleep test % (n)** | **EMM [95% CI]** |
| --- | --- | --- | --- | --- |
| Baseline  1 month  3 months  6 months  12 months | 97.0 (676)  84.9 (592)  79.3 (553)  75.6 (527)  79.8 (556) | 17.2 [16.9 to 17.4]  17.7 [17.5 to 17.9]  18.1 [18.0 to 18.3]  18.5 [18.4 to 18.7]  18.4 [18.2 to 18.6] | 96.4 (666)  82.1 (567)  76.4 (528)  75.4 (521)  75.4 (521) | 17.0 [16.8 to 17.2]  17.7 [17.5 to 17.9]  18.2 [18.0 to 18.3]  18.5 [18.3 to 18.6]  18.4 [18.2 to 18.6] |

EMM: Estimated marginal mean, CI: Confidence Interval

Supplementary Table 3: Mean scores and numbers of patients that completed categories of the RAND-36 questionnaire at baseline and 1, 3, 6 and 12 months after surgery

|  | **Pulse oximetry % (n)** | **EMM [95% CI]** | **Sleep test % (n)** | **EMM [95% CI]** |
| --- | --- | --- | --- | --- |
| **Physical functioning**  Baseline  1 month  3 months  6 months  12 months | 95.4 (665)  83.8 (584)  68.1 (475)  75.6 (527)  82.2 (573) | 67.9 [65.6 to 70.1]  76.9 [75.1 to 78.6]  84.7 [83.2 to 86.1]  87.0 [85.6 to 88.3]  88.7 [87.3 to 90.1] | 94.6 (654)  82.2 (568)  75.3 (520)  74.2 (513)  76.6 (529) | 68.5 [66.3 to 70.7]  78.2 [76.6 to 79.9]  84.2 [82.7 to 85.6]  87.6 [86.2 to 88.9]  89.6 [88.1 to 90.1] |
| **Role functioning physical**  Baseline  1 month  3 months  6 months  12 months | 94.5 (659)  83.8 (584)  66.7 (465)  74.2 (517) 81.1 (565) | 69.2 [65.5 to 72.7]  55.4 [51.4 to 59.2]  89.0 [86.8 to 91.1]  94.2 [92.5 to 95.7]  92.9 [90.9 to 94.7] | 94.6 (654)  82.1 (567)  75.0 (518)  73.5 (508)  76.3 (527) | 68.3 [64.6 to 71.8]  57.8 [53.9 to 61.5]  87.9 [85.7 to 89.9]  94.8 [93.1 to 96.2]  92.7 [90.7 to 94.6] |
| **Role functioning emotional**  Baseline  1 month  3 months  6 months  12 months | 94.1 (656)  82.8 (577)  66.4 (463)  74.5 (519)  81.5 (568) | 91.8 [90.0 to 93.5]  90.1 [88.3 to 91.8]  95.9 [94.5 to 97.0]  96.5 [95.2 to 97.6]  95.6 [94.2 to 96.9] | 95.1 (657)  81.2 (561)  73.2 (506)  73.4 (507)  76.6 (529) | 91.4 [89.6 to 93.0]  88.4 [86.4 to 90.2]  95.5 [94.1 to 96.6]  96.6 [95.4 to97.7]  95.2 [93.7 to 96.6] |
| **Energy/fatigue**  Baseline  1 month  3 months  6 months  12 months | 96.4 (672)  85.4 (595)  80.3 (560)  77.6 (541)  83.6 (583) | 51.6 [49.9 to 53.4]  56.5 [54.9 to 58.0]  64.7 [63.2 to 66.2]  69.3 [67.7 to 70.9]  69.0 [67.2 to 70.7] | 97.1 (671)  83.2 (575)  77.0 (532)  75.4 (521)  79.7 (551) | 51.4 [49.7 to 53.0]  56.5 [54.9 to 58.0]  66.0 [64.4 to 67.5]  68.0 [66.3 to 69.6]  69.3 [67.5 to 71.0] |
| **Emotional well-being**  Baseline  1 month  3 months  6 months  12 months | 97.7 (681)  85.2 (594)  80.3 (560)  77.5 (540)  83.8 (584) | 75.7 [74.0 to 77.3]  81.9 [80.7 to 83.2]  84.3 [83.1 to 85.4]  85.1 [83.9 to 86.3]  84.5 [83.1 to 85.8] | 96.7 (668)  82.6 (571)  77.7 (537)  75.8 (524)  79.9 (552) | 76.2 [74.6 to 77.8]  82.2 [80.9 to 83.5]  83.4 [82.2 to 84.6]  83.4 [82.1 to 84.7]  84.2 [82.8 to 85.6] |
| **Social functioning**  Baseline  1 month  3 months  6 months  12 months | 94.1 (656)  82.5 (575)  65.1 (454)  72.6 (506)  79.6 (555) | 78.7 [76.7 to 80.7] 80.8 [79.0 to 82.5]  91.9 [90.6 to 93.1]  92.8 [91.5 to 93.9]  92.7 [91.4 to 94.0] | 95.1 (657)  79.3 (548)  72.9 (504)  73.8 (510)  76.0 (525) | 78.9 [76.9 to 80.8]  79.1 [77.2 to 80.9]  90.1 [88.8 to 91.4]  93.0 [91.7 to 94.1]  92.6 [91.2 to 93.9] |
| **Pain**  Baseline  1 month  3 months  6 months  12 months | 94.8 (661)  84.1 (586)  67.1 (468)  73.7 (514)  81.3(567) | 72.7 [70.4 to 74.9]  71.3 [69.2 to 73.3]  86.2 [84.7 to 87.6]  87.7 [86.2 to 89.1]  89.0 [87.4 to 90.4] | 95.7 (661)  81.3 (562)  74.1 (512)  73.8 (510)  77.0 (532) | 71.4 [69.2 to 73.6]  71.8 [69.8 to 73.8]  86.2 [84.7 to 87.6]  86.6 [85.0 to 88.0]  87.6 [86.0 to 89.1] |
| **General health**  Baseline  1 month  3 months  6 months  12 months | 97.0 (676)  84.6 (590)  68.0 (474)  75.8 (528)  83.1 (579) | 56.4 [54.9 to 57.9]  66.0 [64.7 to 67.4]  68.8 [67.4 to 70.2]  71.2 [69.8 to 72.6]  73.2 [71.6 to 74.7] | 96.1 (664)  83.4 (576)  76.1 (526)  75.0 (518)  79.5 (549) | 57.4 [55.9 to 58.9]  67.0 [65.6 to 68.3]  69.3 [68.0 to 70.6]  72.6 [71.2 to 74.0]  73.9 [72.4 to 75.5] |
| **Health change**  Baseline  1 month  3 months  6 months  12 months | 99.4 (693)  88.8 (619)  71.0 (495)  79.2 (552)  85.4 (595) | 44.5 [43.1 to 45.9]  70.4 [68.7 to 72.1]  82.2 [80.3 to 84.2]  89.0 [87.0 to 91.0]  90.3 [88.2 to 92.4] | 99.4 (687)  86.4 (597)  79.2 (547)  78.0 (539)  80.9 (559) | 46.5 [45.1 to 47.9]  73.8 [72.1 to 75.5]  85.0 [83.1 to 86.8]  89.8 [87.7 to 91.8]  89.7 [87.6 to 91.9] |

EMM: Estimated marginal mean, CI: Confidence Interval

Supplementary Table 4: Mean total weight loss 1, 3, 6 and 12 months after surgery

|  | **Pulse oximetry % (n)** | **EMM [95% CI]** | **Sleep test % (n)** | **EMM [95% CI]** |
| --- | --- | --- | --- | --- |
| 1 month  3 months  6 months  12 months | 97.7 (681)  97.1 (677)  95.7 (667)  93.7 (653) | 9.0 [8.6 to 9.5]  17.9 [17.5 to 18.3]  26.5 [26.0 to 26.9]  34.0 [33.6 to 34.4] | 97.1 (671)  94.8 (655)  90.2 (623)  91.5 (632) | 9.7 [9.3 to 10.1]  18.1 [17.7 to 18.5]  25.9 [25.5 to 26.3]  32.4 [32.0 to 32.8] |

EMM: Estimated marginal mean, CI: Confidence Interval
